# Evaluation of breast cancer symptoms and risk factors in women: the case of Türkiye

**DOI:** 10.1590/1980-220X-REEUSP-2025-0240en

**Published:** 2025-12-19

**Authors:** Sümeyye Ahi, Muradiye Karasu Ayata, Merve Seyhan

**Affiliations:** 1 Kırşehir Ahi Evran University, Faculty of Health Sciences, Department of Midwifery, Kırşehir, Türkiye.; 2 Kırşehir Ahi Evran University, Faculty of Health Sciences, Department of Nutrition and Dietetics, Kırşehir, Türkiye.

**Keywords:** Breast Cancer, Risk Factors, Symptom Assessment, Breast Self-Examination, Câncer de Mama, Risk Factors, Avaliação de Sintomas, Autoexame de Mama

## Abstract

**Objective:**

This study aimed to evaluate the frequency of breast cancer symptoms and the distribution of risk factors among women aged 18 years and above.

**Methods:**

This descriptive study was conducted with 380 women who were registered at a Family Health Center in a central city of Türkiye. Data was collected through face-to-face interviews. Descriptive statistics, as well as the independent t-test, chi-square test, and Fisher's Exact test were used in the analysis.

**Results:**

The mean age of participants was 44.5 ± 15.5 years. Breast cancer symptoms such as breast or armpit pain, nipple changes, and swelling were observed in 32.1% of the participants. Among those with symptoms, 61.5% did not know how to perform a breast self-examination. Additionally, 20.3% of the participants had never participated in screenings, most commonly due to long waiting times for appointments. Short breastfeeding duration, smoking, and a lack of knowledge about self-examination were prominent risk factors.

**Conclusion:**

Despite the prevalence of symptoms, low levels of knowledge about self-examination and low participation in screenings are noteworthy. From the results of this study, promoting early detection, expanding health education, and improving access to screening services are recommended.

## RESUMO

**Objetivo:**

Este estudo teve como objetivo avaliar a frequência dos sintomas de câncer de mama e a distribuição dos fatores de risco entre mulheres com idade igual ou superior a 18 anos.

**Métodos:**

Trata-se de um estudo descritivo realizado com 380 mulheres cadastradas em uma Unidade de Saúde da Família em uma cidade central da Turquia. Os dados foram coletados por meio de entrevistas presenciais. Na análise, foram utilizadas estatísticas descritivas, bem como o teste t para amostras independentes, o teste do qui-quadrado e o teste exato de Fisher.

**Resultados:**

A idade média das participantes foi de 44,5 ± 15,5 anos. Sintomas relacionados ao câncer de mama, como dor na mama ou axila, alterações no mamilo e inchaço, foram observados em 32,1% das participantes. Entre aquelas que apresentaram sintomas, 61,5% não sabiam como realizar o autoexame das mamas. Além disso, 20,3% das mulheres nunca haviam participado de exames de rastreamento, sendo o longo tempo de espera para consultas a razão mais frequentemente relatada. A curta duração da amamentação, o tabagismo e a falta de conhecimento sobre o autoexame destacaram-se como fatores de risco predominantes.

**Conclusão:**

Apesar da presença de sintomas, observou-se baixo nível de conhecimento e prática do autoexame das mamas, bem como baixa participação em programas de rastreamento. Com base nos resultados deste estudo, recomenda-se a promoção da detecção precoce, a ampliação da educação em saúde e a melhoria do acesso aos serviços de rastreamento.

## INTRODUCTION

Breast cancer is the most commonly diagnosed cancer among women^(1)^. According to the World Health Organization (2025), it ranks first among female cancers in 157 of 185 countries worldwide^(1)^. Accounting for approximately 15% of all cancer-related deaths, its age-standardized incidence rate is reported as 46.8 per 100,000 people, with a mortality rate of 12.7 per 100,000^(2)^. Globally, breast cancer is responsible for an estimated 670,000 deaths annually, and by 2040, the number of new cases is projected to exceed 3 million, with over 1 million deaths^(3)^. These figures underscore the substantial global burden of the disease and the critical importance of early detection and the implementation of preventive strategies^(4)^.

Advances in screening and treatment have led to a marked decline in mortality, particularly in high-income countries, where age-standardized mortality rates decreased by approximately 40% between the 1980s and 2020^(1)^. Nevertheless, breast cancer remains the second leading cause of cancer-related deaths among women and the leading cause among Black and Hispanic women^(4,5)^.

Early detection rates depend largely on the accessibility and utilization of screening programs. In low- and middle-income countries, screening coverage remains inadequate, with mammography utilization rates ranging between 10% and 50%, and limited uptake of low-cost methods such as clinical breast examinations and breast self-examinations (BSE)^(6)^. Populationbased, organized mammography programs have been shown to reduce mortality by 25–40%^(7)^. Socio-demographic factors, including age, education, income, residence, and health insurance status, along with personal concerns, religious beliefs, stigma, and financial barriers significantly influence participation^(6,8)^.

In Türkiye, the national mammography screening program is implemented through Cancer Early Diagnosis, Screening, and Training Centers^(9)^. However, participation rates vary regionally and remain suboptimal, with national survey data indicating coverage rates of 55% in 2019 and 53% in 2022 among women aged 40–69 years^(10)^. Field studies further reveal that only 7.1% of women have ever undergone mammography and that 63.7% are unaware of its necessity^(11)^, highlighting gaps in both awareness and access.

Risk factors for breast cancer encompass hormonal, metabolic, lifestyle, and genetic components. Incidence increases with age, with the highest risk in women over 50 years old^(12,13,14)^. In low- and middle-income countries, diagnosis often occurs at younger ages 45–52 years in Sub-Saharan Africa, 36–56 years in the Middle East, and 49–53 years in Latin America compared to 55–76 years in high-income countries, where most cases are postmenopausal^(3,13,15,16,17)^. Prolonged estrogen exposure, early menarche (<12 years), late menopause (≥55 years), nulliparity, first childbirth after age 30 years old, and obesity particularly with high body fat despite a normal BMI are established risk factors^(12,18,19)^. Weight gain before menopause can increase risk by 12–15%^(12)^, while smoking initiation before the age of 15 years or prior to first childbirth further elevates risk^(20)^. BRCA1 and BRCA2 mutations, a family history of breast or ovarian cancer, low socioeconomic status, delayed diagnosis, limited healthcare access, and inadequate infrastructure also adversely affect incidence and prognosis^(12,17)^.

Between 2002 and 2019, breast cancer incidence in Türkiye increased by 74.8%^(21)^. This sharp rise, despite the expansion of screening programs, suggests that early detection remains insufficient and that public knowledge of risk factors and early symptoms is limited. Therefore, this study aimed to assess women’s awareness of breast cancer symptoms and risk factors in Türkiye, providing evidence to improve screening program effectiveness and to inform targeted early detection strategies. One of the strengths of this study is that it uses primary data collected from a community-based sample, thereby providing up-to-date and context-specific evidence on women’s awareness levels.

Research Questions

1. What is the frequency of breast cancer symptoms among participants?
2. What is the distribution of breast cancer risk factors among participants?
3. Is there a difference between participants’ mean age and the presence of breast cancer symptoms?
4. Is there a relationship between breast cancer risk factors and the presence of symptoms among participants?

## METHOD

### Design of Study

Descriptive type of research.

### Population and Local

The study population consisted of approximately 10,000 women aged between 18 and 65 years who were registered at a Family Health Center (FHC) located in the city center of Kırşehir, a province situated near the capital of Türkiye. This center operates under the public health system and provides free healthcare services to all applicants.

### Selection Criteria

Women aged 18 years and above who could understand and read Turkish were included in the study. Women that have been diagnosed with any psychiatric disorder that could prevent them from answering the survey questions were excluded from the study.

### Sample Definition

According to the 2022 Health Statistics Yearbook published by the Ministry of Health, the prevalence of women who have never performed a breast self-examination is 52.4%^(21)^. Based on this statistic, the minimum required sample size was calculated using the Epi Info 7.2.6.0 program, assuming a 95% confidence level, a 52.4% prevalence rate, a 0.05 margin of error, and a design effect of 1. As a result, the minimum sample size was determined to be 369 women. Considering potential data loss, it was decided to include 380 women in the study and data collection was completed with a total of 380 participants.

### Data Collection

Data collection began after obtaining approval from the ethics committee and the relevant institution. The selected Family Health Center (FHC) was chosen due to its advantageous location along public transportation routes such as buses and minibuses, its relatively high number of family physicians (six in total), the large population it serves, and the high number of applicants. The data for the study were collected between December 2, 2024, and March 10, 2025 during working hours through face-to-face interviews conducted by the researchers.

### Data Collection Tools

The questionnaire used in this study was developed by the researchers based on a comprehensive literature review. Questions addressing breast cancer risk factors and symptoms were examined, drawing on both national and international studies^(1,2,3,4,5,6,7,8,9,10,11,12,13,14,15,16,17,18,19,20,21)^. Within this framework, a 45-item form was designed with the following distribution:

*Sociodemographic characteristics (19 items):* Age, education level, marital status, family type, occupation, income level, place of residence, number of children, breastfeeding status, history of chronic disease, smoking habits, and menopausal status.

*Breast cancer risk factors (8 items):* Early menarche (<12 years), late menopause (≥55 years), use of birth control/hormones, infertility, age at first birth (≥30), obesity, and high-fat diet. Responses were recorded as “Yes/No” items.

*Breast cancer symptoms (8 items):* Nipple retraction, discharge or bleeding, lump/swelling in the breast or armpit, and changes in breast shape or color, assessed through “Yes/No” items.

*Breast cancer awareness (10 items):* Participation in screening programs, knowledge of breast self-examination (BSE), and mammography use. Reasons for not attending screenings (e.g., lack of time and lack of information) and certain health behaviors (such as fatty/sweet food consumption) were also included.

This structure enabled a comprehensive evaluation of both participants’ sociodemographic characteristics and their knowledge and attitudes toward breast cancer.

Prior to data collection, the questionnaire was piloted with 20 women to assess clarity. Unclear items were revised, after which data collection commenced.

As the data were collected in a family health center, time constraints posed a notable challenge. Many women preferred to leave immediately after their medical appointments and were reluctant to complete the questionnaire. This situation reduced participation rates and posed difficulties in the data collection process.

### Data Analysis

The research data were analyzed using IBM SPSS Statistics version 20.0 (IBM Corp., Armonk, NY, USA). In descriptive analyses, continuous variables (age, marital duration, number of living children, and total breastfeeding duration) were summarized as mean, standard deviation, minimum, and maximum values, while categorical variables (place of residence, employment status, participation in cancer screening programs, etc.) were expressed as frequencies and percentages.

Normality assumptions were evaluated using skewness and kurtosis coefficients. Values within ±1 (0.286; –0.760) were considered indicative of a normal distribution. For the independent samples t-test, Levene’s test was applied to assess the homogeneity of variances, and this assumption was met (p > 0.05). Statistical significance was set at p < 0.05 for all analyses.

In line with the research questions, the distribution of participants’ breast cancer symptoms and risk factors is presented in tables as counts and percentages. Differences in mean age according to the presence of breast cancer symptoms were analyzed using the independent samples t-test. Associations between breast cancer risk factors and the presence of symptoms were examined using the Pearson Chi-Square test, with Fisher’s Exact test applied when the expected cell count was <5. Furthermore, the relationships between participants’ breast cancer screening participation, knowledge of BSE, and the presence of breast cancer symptoms were also analyzed using the Pearson Chi-Square test.

### Ethical Aspects

Ethical approval for the study was obtained from the Ethics Committee for Scientific Research and Publication at Kırşehir Ahi Evran University, Faculty of Social and Human Sciences (Decision No: 2024/13/06, dated 20.11.2024). In addition, institutional permission was granted by the Kırşehir Provincial Health Directorate (Document No: E-42884709-020-259412501). Prior to data collection, all participants were informed about the purpose and procedures of the study, and both written and verbal informed consent were obtained. The study was conducted in accordance with the ethical principles outlined in the Declaration of Helsinki.

## RESULTS

Table 1 presents the sociodemographic information of the participants. According to the table, the average age of the participants was 44.5 ± 15.5 years, the average number of living children was 2.8 ± 1.5, and the breastfeeding duration was 19.2 ± 7.8 months. Of the participants, 29.2% completed primary school, 54.7% lived in urban centers, and 35.8% reported that they had entered menopause (Table 1).

**Table 1:** Sociodemographic Characteristics of the Participants – Kırşehir, Türkiye, 2025.

Variable	Mean ± SD	Min.–max.
Age	44,5 ± 15,5 years	18–85
Marital duration	25,4 ± 15,5 years	1–60
Number of living children	2,8 ± 1,5	1–11
Breastfeeding duration	19,2 ± 7,8 months	1–36
	**n = 380**	**%**
Educational Status	
Illiterate	28	7,4
Primary school graduate	111	29,2
Secondary school graduate	72	18,9
High school graduate	103	27,1
University degree or higher	66	17,4
Religious Affiliation	
Muslim	380	100
Primary Residence	
City center	208	54,7
District	89	23,4
Village	75	19,7
Town	8	2,1
Perceived Income Level	
Less than expenses	43	11,3
Equal to expenses	237	62,4
More than expenses	100	26,3
Employment Status		
Employed	114	30,0
Unemployed	266	70,0
Marital Status		
Married	257	67,4
Single	61	16,1
Divorced	15	3,9
Widowed	47	12,4
Family Type		
Nuclear family	91	23,9
Extended family	289	76,1
Presence of Chronic Illness		
Yes	160	42,1
No	220	57,9
Presence of Living Children		
Yes	301	79,2
No	79	20,8
Menopausal Status	
Yes	136	35,8
No	244	64,2

Table 2 presents the distribution of participants based on their engagement in breast cancer screening and the presence of breast cancer symptoms. According to the findings, four out of five participants (79.7%) had participated in breast cancer screening. Among those who had not, the majority (61.4%) stated that the reason was the long waiting time to see a doctor. Breast cancer symptoms were reported by 32.1% of the participants. The three most common symptoms were: pain in one of the breasts or armpits (28.3%), changes in the shape, size, or color of the breast or nipple (15.5%), and partial or complete swelling of the breast or armpit (13.9%) (Table 2).

**Table 2:** Distribution of participation in breast cancer screening and presence of breast cancer symptoms – Kırşehir, Türkiye, 2025.

Variable (n = 380)		n^*^	%
Participation in breast cancer screening	Yes	303	79,7
	No	77	20,3
Reasons for not participating in screening (n = 77)^*^	I do not want to go to the doctor for screening	2	2,0
	Waiting for a doctor appointment takes too long	62	61,4
	I am too busy and have no time for screening	37	36,6
Presence of breast cancer symptoms	Yes	122	32,1
	No	258	67,9
Types of breast cancer symptoms reported (n = 122)^*^	Pain in one of the breasts or armpits	53	28,3
	Changes in the shape, size, or color of the breast or nipple	29	15,5
	Partial or complete swelling in the breast or armpit	26	13,9
	Lump or thickening in the breast	21	11,2
	Nipple discharge or bleeding	17	9,1
	Lump or thickening in the armpit	16	8,6
	Dimpling or puckering of the nipple	15	8,0
	Retraction of the nipple	10	5,3

* Multiple responses were allowed.

Table 3 compares the participants’ engagement in breast cancer screening and their knowledge of BSE with the presence of breast cancer symptoms. According to the findings, 61.5% of those with symptoms stated that they did not know how to perform BSE, and this difference was statistically significant (p < 0.05) (Table 3).

**Table 3:** Comparison of participation in breast cancer screening and knowledge of bse with the presence of breast cancer symptoms – Kırşehir, Türkiye, 2025.

Variables		Breast cancer symptom status				*χ*2	p
		Yes		No			
		n	%	n	%		
Participation in breast cancer screening	Yes	92	75,4	211	81,8	2,082^*^	0,149
	No	30	24,6	47	18,2		
Knowledge of BSE	Yes	21	17,2	83	32,2	11,606^*^	0,003^**^
	No	75	61,5	143	55,4		
	Not sure	26	21,3	32	12,4		

* Pearson Chi-Square

** p < 0,05.

Table 4 presents the comparison between the participants’ mean age and the presence of specific breast cancer symptoms. According to the findings, participants with nipple retraction and those with dimpling or puckering of the nipple had significantly higher mean ages (p < 0.05) (Table 4).

**Table 4:** Comparison of participants’ mean age with breast cancer symptoms – Kırşehir, Türkiye, 2025.

Symptoms		n	Age mean ± SD	t-test	p-value
Nipple discharge or bleeding	Yes	17	40,9 ± 15,5	0,952	0,342
	No	363	44,6 ± 15,5		
Partial or full swelling in breast/armpit	Yes	26	43,3 ± 13,9	0,388	0,698
	No	354	44,5 ± 15,7		
Changes in breast/nipple shape, size, or color	Yes	29	45,5 ± 15,0	0,372	0,710
	No	351	44,4 ± 15,6		
Pain in breast or armpit	Yes	53	45,9 ± 13,9	0,716	0,475
	No	327	44,2 ± 15,8		
Nipple retraction	Yes	10	57,1 ± 12,8	**2,629**	0,009^*^
	No	370	44,1 ± 15,5		
Lump or thickening in armpit	Yes	16	42,4 ± 14,7	0,529	0,597
	No	364	44,5 ± 15,6		
Dimpling or puckering of the nipple	Yes	15	55,2 ± 18,5	**2,757**	0,006^*^
	No	365	44,0 ± 15,3		
Lump or thickening in the breast	Yes	21	41,6 ± 15,1	0,859	0,391
	No	359	44,6 ± 15,6		

* p < 0.05, t = Independent samples t-test.

This table presents the comparison between the presence of breast cancer symptoms and various risk factors among participants. According to the findings, 56.6% of women with symptoms had breastfed their children for less than 24 months, and this difference was statistically significant (p < 0.05). Additionally, only 17.2% of symptomatic women reported knowing how to perform BSE, while 61.5% stated they did not, which was also significant (p < 0.05). Although higher symptom rates were observed in women with first-degree family history of breast cancer, radiation exposure, hormonal contraceptive use, hormone therapy, early menarche, late menopause, infertility history, first birth after age 30, and those who did not participate in screening, these differences were not statistically significant (p > 0.05) (Table 5).

**Table 5:** Comparison of breast cancer symptoms with risk factors – Kırşehir, Türkiye, 2025.

Risk factor		Breast cancer symptom status				*χ* ^2^	p-value
		Yes		No			
		n	%	n	%		
Having living children	Yes	105	86,1	196	76,0	5,128^*^	0,024^***^
	No	17	13,9	62	24,0		
First birth after age 30	Yes	8	6,6	13	5,0	0,366^*^	0,545
	No	114	93,4	245	95,0		
Duration of breastfeeding	Breastfeeding <24 months	69	56,6	108	41,9	7,237^*^	0,027^***^
	Breastfeeding >24 months	31	25,4	85	32,9		
	Never breastfed	22	18,0	65	25,2		
Degree of relative diagnosed with cancer	First-degree relative	20	47,6	13	34,2	1,480^*^	0,224
	Second- or third-degree relative	22	52,4	25	65,8		
Radiation exposure status	Yes	5	4,1	9	3,5	0,085^**^	0,775
	No	117	95,9	249	96,5		
Hormonal contraceptive use	Yes	48	39,3	94	36,6	0,271^*^	0,603
	No	74	60,7	163	63,4		
Hormone therapy status	Yes	27	22,1	40	15,6	2,451^*^	0,117
	No	95	77,9	217	84,4		
Early menarche (<12 years)	Yes	24	19,7	40	15,6	0,995^*^	0,319
	No	98	80,3	217	84,4		
Late menopause (>55 years)	Yes	11	9,0	11	4,3	3,394^*^	0,065
	No	111	91,0	246	95,7		
History of infertility	Yes	5	4,1	3	1,2	3,463^**^	0,117
	No	117	95,9	255	98,8		
High-fat diet	Yes	48	39,3	113	43,8	0,673^*^	0,412
	No	74	60,7	145	56,2		
Overweight	Yes	12	9,8	30	11,6	0,271^*^	0,603
	No	110	90,2	228	88,4		
Smoking status	Yes	52	42,6	109	42,2	0,005^*^	0,945
	No	70	57,4	149	57,8		

* Pearson Chi-Square

** Fisher Exact Test

*** p < 0,05.

## DISCUSSION

This study revealed that nearly one in three women (32.1%) reported experiencing breast cancer symptoms, a finding that signals not only a high symptom burden but also insufficient awareness and suboptimal adoption of early detection practices in the study region. Global evidence shows that breast cancer incidence continues to rise, and access to early diagnosis remains particularly limited in low-income regions^(22)^. In low- and middle-income countries (LMICs), mammography utilization rates generally range from 10% to 50%, while the uptake of low-cost methods such as clinical breast examination (CBE) and BSE also remains low. Although screening rates have improved since 2010 and 2016, accessibility remains inadequate^(6)^. In our study, more than one-fifth of participants (20.3%) had never engaged in any screening program, and the majority of symptomatic women (61.5%) did not know how to perform a BSE, highlighting a strong link between individual awareness and participation in early detection practices.

Evidence from Türkiye and other LMICs consistently identifies knowledge deficits as a primary barrier to screening uptake. In one study of women over the age of 40, 99.2% cited lack of information and education as the reasons for not undergoing mammography^(23)^. A national survey found that 13% of women who did not attend screenings were unaware of the necessity of BSE, and 10% did not know how to perform it. Similar patterns emerge across the Middle East and North Africa: in Egypt, women knowledgeable about and regularly practicing BSEs were significantly less likely to be diagnosed at an advanced stage^(24)^, while in Iran, a multicenter study demonstrated that BSE knowledge and practice reduced the risk of late-stage diagnosis^(25)^. In Jordan, however, only 25.5% of women regularly practiced BSEs, 10.5% underwent regular CBEs, and just 11.1% reported consistent mammography use^(8)^. Barriers identified included personal concerns, perceived examination-related discomfort, low health motivation, religious beliefs, societal norms, insufficient social support, stigma-related fears, and competing work–family responsibilities. While education level was positively associated with knowledge, cultural and social constraints often hindered the translation of awareness into consistent screening behavior. These findings indicate that improving knowledge alone may be insufficient unless interventions address underlying cultural norms and social support systems.

Beyond individual awareness, systemic barriers—particularly long waiting times for medical appointments—emerged as a significant deterrent to screening participation. In our study, 61.4% of women who had never undergone screening cited long wait times as the primary reason. For disadvantaged populations, extended delays and restricted healthcare access compound the risk of delayed diagnosis. This aligns with prior research showing that women who encounter healthcare access difficulties are less likely to participate in screening programs, ultimately increasing the likelihood of late-stage presentation^(26)^. Addressing these barriers requires more than health education; it demands structural reforms, including increased capacity for screening services, reduced appointment delays, and equitable resource distribution. Comprehensive interventions targeting both health literacy and healthcare system accessibility are essential to narrowing disparities and improving early detection outcomes.

The average age of participants was 44.5 ± 15.5 years, and symptoms such as nipple retraction, dimpling, and indentation, commonly associated with advanced stages of breast cancer, were significantly more prevalent among older women (p < 0.05). This finding demonstrates that age is not only a risk factor but also influences the severity and nature of symptoms. One study identified women aged 50–69 years as the most affected group, with incidence increasing with age^(26)^. Additionally, breast cancer in younger women tends to present more aggressive characteristics and is often diagnosed at a later stage due to delays in recognition^(27)^. In Africa, the mean age at diagnosis is 48.7 years, which is lower than in high-income countries^(15)^. In low- and middle-income countries, the age at diagnosis tends to be younger^(16)^. For example, in Sub-Saharan Africa, it ranges from 45 to 52 years, in the Middle East from 36 to 56 years, and in Latin America from 49 to 53 years. In China, the highest disease burden is observed in the 50–59 age group, with a marked increase in incidence among women aged over 40^(13)^. Similarly, in high-income countries, the highest risk is reported among women aged 50 years and older, with the risk continuing to rise in this age group^(12)^. A large multicenter meta-analysis of 44 studies reported a mean diagnostic age ranging from 55 to 76.4 years, with a significant increase in risk after menopause^(3)^. In Brazil, the mean age at diagnosis was reported as 55 years^(17)^. Collectively, these findings demonstrate that age influences not only the level of breast cancer risk but also the presentation of symptoms, disease progression, and the timing of diagnosis. Therefore, screening programs should incorporate both age and symptom characteristics into risk stratification.

Early menarche and late menopause, indicative of prolonged estrogen exposure, are considered significant risk factors for breast cancer^(28,29)^. Research has shown that menarche after age 15 reduces the risk of breast cancer by 23% compared to menarche before age 12^(30)^. Studies on hormonal factors indicate that prolonged exposure to estrogen increases the risk of breast cancer, a condition typically observed in women with early onset of menstruation (< 12 years) and late menopause (≥ 55 years)^(12,18)^. In addition, having no children or having a first childbirth after the age of 30 are also considered additional risk factors for breast cancer^(12)^. Although our study found higher rates of breast cancer symptoms among women with early menarche and late menopause, the differences were not statistically significant (p > 0.05). This may be due to a limited sample size in the late menopause group or the overall age distribution. However, the p-value for late menopause (p = 0.065) approached statistical significance, suggesting a clinically meaningful trend. These results align with the existing literature, indicating that prolonged hormonal exposure may increase the likelihood of breast cancer symptoms.

Our study examined reproductive factors such as age at first birth, number of pregnancies, and number of living children in relation to the presence of breast cancer symptoms. Although the rate of symptoms was lower among women who gave birth before age 30, the difference was not statistically significant (p > 0.05). Nevertheless, existing literature consistently supports the protective effect of early childbirth and high parity against breast cancer. For example, women who gave birth before age 30 have been shown to have a lower risk of developing luminal subtypes of breast cancer^(28)^. Likewise, first childbirth before age 20 is considered protective, while childbirth after age 35 is considered a risk factor^(30)^. The protective mechanism is thought to involve hormonal regulation during pregnancy and reduced estrogen exposure. A higher number of pregnancies has also been found to reduce breast cancer risk. Women who never give birth are at increased risk, which becomes more pronounced when the first pregnancy occurs at an older age. Although no statistically significant differences were found in our study, these findings underscore the importance of monitoring reproductive factors in relation to breast cancer symptoms and highlight the need for studies with larger sample sizes.

A significant finding of our study was that women who breastfed for less than 24 months were more likely to report breast cancer symptoms (p < 0.05). This finding supports the protective role of breastfeeding against breast cancer. The duration and timing of breastfeeding, along with parity, may contribute to this protective effect. A systematic review found that long-term breastfeeding provides significant protection against various subtypes of breast cancer^(31)^. Another study emphasized that breastfeeding supports hormonal regulation and mammary gland differentiation, which helps prevent cancer^(28)^. A metaanalysis showed that each 12-month period of breastfeeding reduces breast cancer risk by 4.5%, and each additional birth reduces the risk by approximately 7%^(32)^. Another study reported that breastfeeding can reduce the risk of breast cancer by up to 16%^(33)^. The protective effect is particularly pronounced among women who gave birth at a young age and breastfed for longer durations. The fact that women with symptoms in our study breastfed for less than 24 months is consistent with this literature. Therefore, breastfeeding plays a critical role not only in infant health but also in reducing long-term breast cancer risk. Awareness and support programs promoting longer breastfeeding durations can provide substantial health benefits at both individual and population levels.

Our study did not find a statistically significant association between hormone therapy or hormonal contraceptive use and the presence of breast cancer symptoms (p > 0.05). However, the existing literature suggests that hormonal exposure may increase breast cancer risk. For instance, postmenopausal hormone replacement therapy (HRT) is associated with an elevated risk. A study in the United Kingdom reported a relative risk of 1.66 for breast cancer among HRT users^(34)^. Another study found that long-term use of combined estrogen-progestogen therapy significantly increased the risk, which declined after discontinuation^(35)^. Hormonal contraceptives have also been associated with increased breast cancer risk, especially with prolonged use. A large cohort study reported that the use of hormonal contraceptives increased the risk of breast cancer by 1.20 times, and that a dose-response relationship existed based on duration^(36)^. Another study identified oral contraceptive use as a significant risk factor (OR = 1.16)^(33)^. The lack of significant findings in our study may result from missing data on the duration and type of hormone therapy used. These findings underscore the importance of personalized prevention and treatment approaches that consider individual risk factors and histories of hormonal exposure.

Obesity, diet, smoking, and alcohol consumption are among the lifestyle factors shown in epidemiological cohort and case-control studies to influence breast cancer risk and prognosis^(18)^. An increasing body of evidence supports the link between smoking and breast cancer risk. A systematic review showed that smoking significantly increases the risk of breast cancer and that the risk rises proportionally with the amount smoked^(37)^. Early exposure to smoking may induce changes in breast tissue. Studies have found a significant association between early smoking initiation and breast cancer risk^(38)^. Adolescents who smoke, especially those with a family history of breast cancer, are at heightened risk^(39)^. Other research has shown that nicotine alters the tumor microenvironment, promotes metastasis, and contributes to chemotherapy resistance^(40,41)^. The effect of smoking on breast cancer varies by frequency, cessation duration, fertility status, and cancer subtypes^(42)^. However, there was no significant relationship between smoking status and breast cancer symptoms in our study. This result may be due to missing data on smoking duration, intensity, passive exposure, or variability in symptom recognition among the participants.

However, it is important to interpret these findings in light of certain limitations. This study was conducted in a single city, potentially limiting the generalizability of the results to other regions of Türkiye. The sample included only female participants; therefore, the findings cannot be generalized to men, and data on male breast cancer—although rare—were not captured. Furthermore, as the data were self-reported, recall and reporting bias may have influenced the results.

## CONCLUSION

This study demonstrated that breast cancer symptoms are prevalent among women and that participation in screening programs remains insufficient. Symptoms were more frequently reported by women who breastfed for a shorter duration, lacked knowledge of BSE, and had limited access to healthcare services. Factors such as age, breastfeeding duration, and individual awareness were found to significantly influence the occurrence of symptoms. Although no statistically significant associations were observed between hormonal factors or reproductive history and symptom presence, the trends identified suggest clinically relevant patterns. These findings underscore the need to enhance awareness initiatives, ensure accessible and timely screening services, and develop preventive strategies tailored to individual risk profiles in the fight against breast cancer.

## Data Availability

The data file can be provided by the author via email upon reasonable request from readers.
