# Supplementary material for: Evaluation of breast cancer symptoms and risk factors in women: the case of Türkiye
Source: Rev Esc Enferm USP. 2025 Dec 19;59:e20250240. doi: 10.1590/1980-220X-REEUSP-2025-0240en (PMC12794627; doi:10.1590/1980-220X-REEUSP-2025-0240en)
Supplement: Translation

# Translation: Evaluación de los síntomas y factores de riesgo del cáncer de mama en mujeres: el caso de Turquía

**DOI:** 10.1590/1980-220X-REEUSP-2025-0240es

**Authors:** Sümeyye Ahi, Muradiye Karasu Ayata, Merve Seyhan

**Affiliations:** 1 Kırşehir Ahi Evran University, Faculty of Health Sciences, Department of Midwifery, Kırşehir, Türkiye.; 2 Kırşehir Ahi Evran University, Faculty of Health Sciences, Department of Nutrition and Dietetics, Kırşehir, Türkiye.

**Keywords:** Cáncer de Mama, Factores de Riesgo, Evaluación de Síntomas, Autoexamen de Mamas

## Abstract

**Objetivo:**

Este estudio tuvo como objetivo evaluar la frecuencia de los síntomas de cáncer de mama y la distribución de los factores de riesgo entre mujeres de 18 años o más.

**Métodos:**

Se realizó un estudio descriptivo con 380 mujeres inscritas en un Centro de Salud Familiar en una ciudad del centro de Turquía. Los datos se recopilaron mediante entrevistas cara a cara. Para el análisis se utilizaron estadísticas descriptivas, la prueba t de Student para muestras independientes, la prueba de chi-cuadrado y la prueba exacta de Fisher.

**Resultados:**

La edad media de las participantes fue de 44,5 ± 15,5 años. Los síntomas relacionados con el cáncer de mama, como dolor en el seno o la axila, cambios en el pezón e hinchazón, se observaron en el 32,1% de las mujeres. Entre aquellas que presentaban síntomas, el 61,5% no sabía cómo realizar un autoexamen mamario. Además, el 20,3% de las participantes nunca había participado en programas de detección, siendo los largos tiempos de espera para obtener citas la causa más frecuente. La corta duración de la lactancia materna, el tabaquismo y la falta de conocimiento sobre el autoexamen se identificaron como factores de riesgo predominantes.

**Conclusión:**

A pesar de la presencia de síntomas, los bajos niveles de conocimiento y práctica del autoexamen mamario, así como la escasa participación en los programas de detección, son aspectos preocupantes. A partir de los resultados de este estudio, se recomienda fomentar la detección precoz, ampliar la educación sanitaria y mejorar el acceso a los servicios de cribado.

## INTRODUCCIÓN

El cáncer de mama es el cáncer más diagnosticado entre las mujeres^(1)^. Según la Organización Mundial de la Salud^(1)^, ocupa el primer lugar entre los cánceres femeninos en 157 de los 185 países del mundo. Representa aproximadamente el 15% de todas las muertes relacionadas con el cáncer, y su tasa de incidencia estandarizada por edad es de 46,8 por cada 100 000 personas, con una tasa de mortalidad de 12,7 por cada 100 000^(2)^. A nivel mundial, el cáncer de mama es responsable de aproximadamente 670 000 muertes al año, y se prevé que para 2040 el número de nuevos casos supere los 3 millones, con más de 1 millón de muertes^(3)^. Estas cifras ponen de relieve la considerable carga mundial que supone esta enfermedad y la importancia fundamental de la detección precoz y la aplicación de estrategias preventivas^(4)^.

Los avances en el cribado y el tratamiento han dado lugar a un notable descenso de la mortalidad, especialmente en los países de ingresos altos, donde las tasas de mortalidad estandarizadas por edad disminuyeron aproximadamente un 40% entre la década de 1980 y 2020^(1)^. No obstante, el cáncer de mama sigue siendo la segunda causa principal de muerte por cáncer entre las mujeres y la principal causa entre las mujeres negras e hispanas^(4,5)^.

Las tasas de detección temprana dependen en gran medida de la accesibilidad y la utilización de los programas de detección. En los países de ingresos bajos y medios, la cobertura de la detección sigue siendo insuficiente, con tasas de utilización de la mamografía que oscilan entre el 10% y el 50%, y una aceptación limitada de métodos de bajo costo, como los exámenes clínicos de mama y los autoexámenes de mama (AEM)^(6)^. Se ha demostrado que los programas de mamografía organizados y basados en la población reducen la mortalidad entre un 25% y un 40%^(7)^. Los factores sociodemográficos, como la edad, la educación, los ingresos, el lugar de residencia y la situación en materia de seguro médico, junto con las preocupaciones personales, las creencias religiosas, el estigma y las barreras económicas, influyen significativamente en la participación^(6,8)^.

En Turquía, el programa nacional de detección mediante mamografía se lleva a cabo a través de los Centros de Diagnóstico Precoz, Detección y Formación sobre el Cáncer^(9)^. Sin embargo, las tasas de participación varían según la región y siguen siendo insuficientes, ya que los datos de las encuestas nacionales indican unas tasas de cobertura del 55% en 2019 y del 53% en 2022 entre las mujeres de 40 a 69 años^(10)^. Los estudios de campo revelan además que solo el 7,1% de las mujeres se ha sometido alguna vez a una mamografía y que el 63,7% desconoce su necesidad^(11)^, lo que pone de manifiesto las deficiencias tanto en materia de concienciación como de acceso.

Los factores de riesgo del cáncer de mama abarcan componentes hormonales, metabólicos, de estilo de vida y genéticos. La incidencia aumenta con la edad, siendo el riesgo más elevado en las mujeres mayores de 50 años^(12,13,14)^. En los países de ingresos bajos y medios, el diagnóstico suele producirse a edades más tempranas: entre 45 y 52 años en el África subsahariana, entre 36 y 56 años en Oriente Medio y entre 49 y 53 años en América Latina, en comparación con los 55–76 años en los países de ingresos altos, donde la mayoría de los casos se dan después de la menopausia^(3,13,15,16,17)^. La exposición prolongada al estrógeno, la menarquia precoz (<12 años), la menopausia tardía (≥55 años), la nuliparidad, el primer parto después de los 30 años y la obesidad, especialmente con un alto porcentaje de grasa corporal a pesar de un IMC normal, son factores de riesgo establecidos^(12,18,19)^. El aumento de peso antes de la menopausia puede aumentar el riesgo entre un 12% y un 15%^(12)^, mientras que empezar a fumar antes de los 15 años o antes del primer parto eleva aún más el riesgo^(20)^. Las mutaciones de los genes BRCA1 y BRCA2, los antecedentes familiares de cáncer de mama u ovario, el bajo nivel socioeconómico, el retraso en el diagnóstico, el acceso limitado a la atención médica y la infraestructura inadecuada también afectan negativamente a la incidencia y el pronóstico^(12,17)^.

Entre 2002 y 2019, la incidencia del cáncer de mama en Turquía aumentó un 74,8%^(21)^. Este fuerte aumento, a pesar de la expansión de los programas de detección, sugiere que la detección temprana sigue siendo insuficiente y que el conocimiento público de los factores de riesgo y los síntomas tempranos es limitado. Por lo tanto, el objetivo de este estudio fue evaluar el conocimiento de las mujeres sobre los síntomas y los factores de riesgo del cáncer de mama en Turquía, proporcionando datos para mejorar la eficacia de los programas de detección y fundamentar estrategias de detección temprana específicas. Uno de los puntos fuertes de este estudio es que utiliza datos primarios recopilados a partir de una muestra comunitaria, lo que proporciona datos actualizados y específicos del contexto sobre los niveles de conocimiento de las mujeres.

Preguntas de Investigación

1. ¿Cuál es la frecuencia de los síntomas del cáncer de mama entre las participantes?
2. ¿Cuál es la distribución de los factores de riesgo de cáncer de mama entre las participantes?
3. ¿Existe alguna diferencia entre la edad media de las participantes y la presencia de síntomas de cáncer de mama?
4. ¿Existe alguna relación entre los factores de riesgo de cáncer de mama y la presencia de síntomas entre las participantes?

## MÉTODO

### Diseño del Estudio

Investigación de tipo descriptivo.

### Población y Ubicación

La población del estudio estaba formada por aproximadamente 10 000 mujeres de entre 18 y 65 años registradas en un Centro de Salud Familiar (CSF) situado en el centro de la ciudad de Kırşehir, una provincia cercana a la capital de Turquía. Este centro funciona bajo el sistema de salud pública y ofrece servicios de atención médica gratuitos a todas las personas que lo solicitan.

### Criterios de Selección

Se incluyó en el estudio a mujeres de 18 años o más que podían entender y leer turco. Se excluyó del estudio a las mujeres a las que se les había diagnosticado algún trastorno psiquiátrico que pudiera impedirles responder a las preguntas de la encuesta.

### Definición de la Muestra

Según el Anuario de Estadísticas de Salud de 2022 publicado por el Ministerio de Salud, la prevalencia de mujeres que nunca se han realizado un autoexamen de mama es del 52,4%^(21)^. Basándose en esta estadística, se calculó el tamaño mínimo de la muestra necesario utilizando el programa Epi Info 7.2.6.0, suponiendo un nivel de confianza del 95%, una tasa de prevalencia del 52,4%, un margen de error del 0,05% y un efecto de diseño de 1. Como resultado, se determinó que el tamaño mínimo de la muestra era de 369 mujeres. Teniendo en cuenta la posible pérdida de datos, se decidió incluir a 380 mujeres en el estudio y la recopilación de datos se completó con un total de 380 participantes.

### Recopilación de Datos

La recopilación de datos comenzó tras obtener la aprobación del comité de ética y de la institución pertinente. Se eligió el Centro de Salud Familiar (CSF) seleccionado debido a su ubicación ventajosa junto a las rutas de transporte público, como autobuses y minibuses, su número relativamente alto de médicos de familia (seis en total), la gran población a la que atiende y el elevado número de solicitantes. Los datos para el estudio se recopilaron entre el 2 de diciembre de 2024 y el 10 de marzo de 2025 durante el horario laboral mediante entrevistas presenciales realizadas por los investigadores.

### Herramientas de Recopilación de Datos

El cuestionario utilizado en este estudio fue elaborado por los investigadores basándose en una revisión exhaustiva de la literatura. Se examinaron las preguntas relativas a los factores de riesgo y los síntomas del cáncer de mama, basándose en estudios nacionales e internacionales^(1,2,3,4,5,6,7,8,9,10,11,12,13,14,15 16,17,18,19,20,21)^. En este marco, se diseñó un formulario de 45 preguntas con la siguiente distribución:

*Características sociodemográficas (19 preguntas*): edad, nivel educativo, estado civil, tipo de familia, ocupación, nivel de ingresos, lugar de residencia, número de hijos, lactancia materna, antecedentes de enfermedades crónicas, hábitos de tabaquismo y estado menopáusico.

*Factores de riesgo de cáncer de mama (8 ítems*): menarquia precoz (<12 años), menopausia tardía (≥55 años), uso de anti-conceptivos/hormonas, infertilidad, edad al primer parto (≥30), obesidad y dieta rica en grasas. Las respuestas se registraron como ítems «Sí/No».

*Síntomas de cáncer de mama (8 ítems*): retracción del pezón, secreción o sangrado, bulto/hinchazón en la mama o la axila y cambios en la forma o el color de la mama, evaluados mediante ítems de «Sí/No».

*Conocimiento sobre el cáncer de mama (10 preguntas*): participación en programas de detección, conocimiento sobre la auto-exploración mamaria (AEM) y uso de la mamografía. También se incluyeron los motivos para no acudir a las revisiones (por ejemplo, falta de tiempo y falta de información) y ciertos hábitos de salud (como el consumo de alimentos grasos/dulces).

Esta estructura permitió una evaluación exhaustiva tanto de las características sociodemográficas de las participantes como de sus conocimientos y actitudes hacia el cáncer de mama.

Antes de la recopilación de datos, se realizó una prueba piloto del cuestionario con 20 mujeres para evaluar su claridad. Se revisaron las preguntas poco claras y, a continuación, se inició la recopilación de datos.

Dado que los datos se recopilaron en un centro de salud familiar, las limitaciones de tiempo supusieron un reto notable. Muchas mujeres preferían marcharse inmediatamente después de sus citas médicas y se mostraban reacias a completar el cuestionario. Esta situación redujo las tasas de participación y planteó dificultades en el proceso de recopilación de datos.

### Análisis de Datos

Los datos de la investigación se analizaron utilizando IBM SPSS Statistics versión 20.0 (IBM Corp., Armonk, Nueva York, EE. UU.). En los análisis descriptivos, las variables continuas (edad, duración del matrimonio, número de hijos vivos y duración total de la lactancia materna) se resumieron como valores medios, desviación estándar, mínimos y máximos, mientras que las variables categóricas (lugar de residencia, situación laboral, participación en programas de detección del cáncer, etc.) se expresaron como frecuencias y porcentajes.

Las hipótesis de normalidad se evaluaron utilizando coeficientes de asimetría y curtosis. Los valores dentro de ±1 (0,286; –0,760) se consideraron indicativos de una distribución normal. Para la prueba t de muestras independientes, se aplicó la prueba de Levene para evaluar la homogeneidad de las varianzas, y se cumplió esta hipótesis (p > 0,05). La significación estadística se fijó en p < 0,05 para todos los análisis.

En consonancia con las preguntas de investigación, la distribución de los síntomas y factores de riesgo de cáncer de mama de las participantes se presenta en tablas como recuentos y porcentajes. Las diferencias en la edad media según la presencia de síntomas de cáncer de mama se analizaron mediante la prueba t para muestras independientes. Las asociaciones entre los factores de riesgo de cáncer de mama y la presencia de síntomas se examinaron mediante la prueba chi-cuadrado de Pearson, aplicando la prueba exacta de Fisher cuando el recuento celular esperado era <5. Además, las relaciones entre la participación de las participantes en las pruebas de detección del cáncer de mama, el conocimiento de la auto-exploración mamaria y la presencia de síntomas de cáncer de mama también se analizaron mediante la prueba chi-cuadrado de Pearson.

### Aspectos Éticos

Se obtuvo la aprobación ética para el estudio del Comité de Ética para la Investigación Científica y la Publicación de la Facultad de Ciencias Sociales y Humanas de la Universidad Ahi Evran de Kırşehir (Decisión n.º: 2024/13/06, de fecha 20.11.2024). Además, se obtuvo el permiso institucional de la Dirección Provincial de Salud de Kırşehir (Documento n.º: E-42884709-020-259412501). Antes de la recopilación de datos, se informó a todas las participantes sobre el propósito y los procedimientos del estudio, y se obtuvo su consentimiento informado tanto por escrito como verbalmente. El estudio se llevó a cabo de acuerdo con los principios éticos descritos en la Declaración de Helsinki.

## RESULTADOS

La Tabla 1 presenta la información sociodemográfica de las participantes. Según la tabla, la edad media de las participantes era de 44,5 ± 15,5 años, el número medio de hijos vivos era de 2,8 ± 1,5 y la duración de la lactancia materna era de 19,2 ± 7,8 meses. De las participantes, el 29,2% había completado la educación primaria, el 54,7% vivía en centros urbanos y el 35,8% declaró haber entrado en la menopausia (Tabla 1).

**Tabla 1:** Características sociodemográficas de las participantes – Kırşehir, Turquía, 2025.

Variable	Media ± DE	Mín.–máx.
Edad	44,5 ± 15,5 años	18–85
Duración del matrimonio	25,4 ± 15,5 años	1–60
Número de hijos vivos	2,8 ± 1,5	1–11
Duración de la lactancia materna	19,2 ± 7,8 meses	1–36
	**n = 380**	**%**
Nivel educativo	
Analfabetos	28	7,4
Graduado de primaria	111	29,2
Graduado de secundaria	72	18,9
Graduado de secundaria	103	27,1
Título universitario o superior	66	17,4
Afiliación religiosa	
Musulmana	380	100
Residencia principal	
Centro de la ciudad	208	54,7
Distrito	89	23,4
Pueblo	75	19,7
Población	8	2,1
Nivel de ingresos percibido	
Inferior a los gastos	43	11,3
Igual a los gastos	237	62,4
Más que los gastos	100	26,3
Situación laboral	
Empleado	114	30,0
Desempleado	266	70,0
Estado civil		
Casado	257	67,4
Soltero	61	16,1
Divorciado	15	3,9
Viudo	47	12,4
Tipo de familia		
Familia nuclear	91	23,9
Familia extensa	289	76,1
Presencia de enfermedades crónicas	
Sí	160	42,1
No	220	57,9
Presencia de hijos vivos		
Sí	301	79,2
No	79	20,8
Estado menopáusico		
Sí	136	35,8
No	244	64,2

La Tabla 2 presenta la distribución de las participantes según su participación en las pruebas de detección del cáncer de mama y la presencia de síntomas de cáncer de mama. Según los resultados, cuatro de cada cinco participantes (79,7%) habían participado en las pruebas de detección del cáncer de mama.

**Tabla 2:** Distribución de la participación en las pruebas de detección del cáncer de mama y presencia de síntomas de cáncer de mama –Kıirçehir, Turquía, 2025

Variable (n = 380)		n^*^	%
Participación en el cribado del cáncer de mama	Sí	303	79,7
	No	77	20,3
Razones para no participar en el cribado (n = 77)^*^	No quiero ir al médico para hacerme las pruebas de detección	2	2,0
	La espera para conseguir una cita con el médico es demasiado larga	62	61,4
	Estoy demasiado ocupada y no tengo tiempo para el cribado	37	36,6
Presencia de síntomas de cáncer de mama	Sí	122	32,1
	No	258	67,9
Tipos de síntomas de cáncer de mama notificados (n = 122)^*^	Dolor en uno de los senos o axilas	53	28,3
	Cambios en la forma, el tamaño o el color de la mama o el pezón	29	15,5
	Hinchazón parcial o completa en el seno o la axila	26	13,9
	Bultos o engrosamiento en la mama	21	11,2
	Secreción o sangrado del pezón	17	9,1
	Bultos o engrosamiento en la axila	16	8,6
	Hundimiento o arrugamiento del pezón	15	8,0
	Retracción del pezón	10	5,3

* Se permitieron respuestas múltiples.

Entre las que no lo habían hecho, la mayoría (61,4%) afirmó que el motivo era el largo tiempo de espera para ver al médico. El 32,1% de las participantes informaron síntomas de cáncer de mama. Los tres síntomas más comunes fueron: dolor en uno de los senos o axilas (28,3%), cambios en la forma, el tamaño o el color del seno o el pezón (15,5%) e hinchazón parcial o completa del seno o la axila (13,9%) (Tabla 2).

La Tabla 3 compara la participación de las participantes en las pruebas de detección del cáncer de mama y sus conocimientos sobre la autoexploración mamaria con la presencia de síntomas de cáncer de mama. Según los resultados, el 61,5% de las personas con síntomas afirmaron que no sabían cómo realizar la autoexploración mamaria, y esta diferencia fue estadísticamente significativa (p < 0,05) (Tabla 3).

**Tabla 3:** Comparación de la participación en las pruebas de detección del cáncer de mama y el conocimiento de la autoexploración mamaria con la presencia de síntomas de cáncer de mama – Kırşehir, Turquía, 2025.

Variables		Estado de los síntomas de cáncer de mama				*χ*2	p
		Sí		No			
		n	%	n	%		
Participación en pruebas de detección de cáncer de mama	Sí	92	75,4	211	81,8	2082^*^	0,149
	No	30	24,6	47	18,2		
Conocimiento de AEM	Sí	21	17,2	83	32,2	11,606^*^	0,003^**^
	No	75	61,5	143	55,4		
	No estoy segura	26	21,3	32	12,4		

* Chi cuadrado de Pearson

** p < 0,05.

La Tabla 4 presenta la comparación entre la edad media de las participantes y la presencia de síntomas específicos de cáncer de mama. Según los resultados, las participantes con retracción del pezón y aquellas con hoyuelos o arrugas en el pezón tenían una edad media significativamente mayor (p < 0,05) (Tabla 4).

**Tabla 4:** Comparación de la edad media de las participantes con los síntomas de cáncer de mama - Kirçehir, Turquía, 2025.

Síntomas		n	Edad media ± DE	Prueba t	Valor p
Secreción o sangrado del pezón	Sí	17	40,9 ± 15,5	0,952	0,342
	No	363	44,6 ± 15,5		
Hinchazón parcial o total en el pecho/axila	Sí	26	433 ± 139	0388	0698
	No	354	44,5 ± 15,7		
Cambios en la forma, el tamaño o el color de los senos/pezones	Sí	29	45,5 ± 15,0	0,372	0,710
	No	351	44,4 ± 15,6		
Dolor en el pecho o la axila	Sí	53	45,9 ± 13,9	0,716	0,475
	No	327	44,2 ± 15,8		
Retracción del pezón	Sí	10	57,1 ± 12,8	**2,629**	0,009^*^
	No	370	44,1 ± 15,5		
Bultos o engrosamientos en la axila	Sí	16	42,4 ± 14,7	0,529	0,597
	No	364	44,5 ± 15,6		
Hundimiento o arrugamiento del pezón	Sí	15	55,2 ± 18,5	**2,757**	0,006^*^
	No	365	44,0 ± 15,3		
Bultos o engrosamientos en la mama	Sí	21	41,6 ± 15,1	0,859	0,391
	No	359	44,6 ± 15,6		

* p < 0,05, t = prueba t para muestras independientes.

Esta tabla presenta la comparación entre la presencia de síntomas de cáncer de mama y diversos factores de riesgo entre las participantes. Según los resultados, el 56,6% de las mujeres con síntomas habían amamantado a sus hijos durante menos de 24 meses, y esta diferencia era estadísticamente significativa (p < 0,05). Además, solo el 17,2% de las mujeres sintomáticas afirmaron saber cómo realizar la autoexploración mamaria, mientras que el 61,5% afirmaron que no sabían, lo que también fue significativo (p < 0,05). Aunque se observaron tasas de síntomas más elevadas en las mujeres con antecedentes familiares de primer grado de cáncer de mama, exposición a la radiación, uso de anticonceptivos hormonales, terapia hormonal, menarquia precoz, menopausia tardía, antecedentes de infertilidad, primer parto después de los 30 años y aquellas que no participaron en las pruebas de detección, estas diferencias no fueron estadísticamente significativas (p > 0,05) (Tabla 5).

**Tabla 5:** Comparación de los síntomas del cáncer de mama con los factores de riesgo – Kırşehir, Turquía, 2025.

Factor de riesgo		Estado de los síntomas del cáncer de mama				*χ* ^2^	Valor p
		Si		No			
		n	%	n	%		
Tener hijos vivos	Sí	105	86,1	196	76,0	5,128^*^	0,024^***^
	No	17	13,9	62	24,0		
Primer parto después de los 30 años	Sí	8	6,6	13	5,0	0,366^*^	0,545
	No	114	93,4	245	95,0		
Duración de la lactancia materna	Lactancia materna <24 meses	69	56,6	108	41,9	7,237^*^	0,027^***^
	Lactancia materna >24 meses	31	25,4	85	32,9		
	Nunca amamantó	22	18,0	65	25,2		
Grado de parentesco del familiar diagnosticado con cáncer	Familiar de primer grado	20	47,6	13	34,2	1,480^*^	0,224
	Pariente de segundo o tercer grado	22	52,4	25	65,8		
Estado de exposición a la radiación	Sí	5	4,1	9	3,5	0,085^**^	0,775
	No	117	95,9	249	96,5		
Uso de anticonceptivos hormonales	Sí	48	39,3	94	36,6	0,271^*^	0,603
	No	74	60,7	163	63,4		
Estado de la terapia hormonal	Sí	27	22,1	40	15,6	2,451^*^	0,117
	No	95	77,9	217	84,4		
Menarquia precoz (<12 años)	Sí	24	19,7	40	15,6	0,995^*^	0,319
	No	98	80,3	217	84,4		
Menopausia tardía (>55 años)	Sí	11	9,0	11	4,3	3,394^*^	0,065
	No	111	91,0	246	95,7		
Antecedentes de infertilidad	Sí	5	4,1	3	1,2	3,463^**^	0,117
	No	117	95,9	255	98,8		
Dieta rica en grasas	Sí	48	39,3	113	43,8	0,673^*^	0,412
	No	74	60,7	145	56,2		
Sobrepeso	Sí	12	9,8	30	11,6	0,271^*^	0,603
	No	110	90,2	228	88,4		
Hábitos tabáquicos	Sí	52	42,6	109	42,2	0,005^*^	0,945
	No	70	57,4	149	57,8		

* Chi-cuadrado de Pearson

** Prueba exacta de Fisher

*** p < 0,05.

## DISCUSIÓN

Este estudio reveló que casi una de cada tres mujeres (32,1%) informó haber experimentado síntomas de cáncer de mama, un hallazgo que indica no solo una elevada carga de síntomas, sino también una concienciación insuficiente y una adopción subóptima de las prácticas de detección precoz en la región objeto del estudio. Las pruebas a nivel mundial muestran que la incidencia del cáncer de mama sigue aumentando y que el acceso al diagnóstico precoz sigue siendo especialmente limitado en las regiones de bajos ingresos^(22)^. En los países de ingresos bajos y medios (PIBM), las tasas de utilización de la mamografía oscilan generalmente entre el 10% y el 50%, mientras que la adopción de métodos de bajo costo, como el examen clínico de las mamas (ECM) y el autoexamen de las mamas (AEM), también sigue siendo baja. Aunque las tasas de detección han mejorado desde 2010 y 2016, la accesibilidad sigue siendo insuficiente^(6)^. En nuestro estudio, más de una quinta parte de las participantes (20,3%) nunca había participado en ningún programa de detección, y la mayoría de las mujeres sintomáticas (61,5%) no sabía cómo realizar un autoexamen de mama, lo que pone de relieve la estrecha relación entre la concienciación individual y la participación en prácticas de detección precoz.

Los datos de Turquía y otros países de ingresos bajos y medios identifican sistemáticamente la falta de conocimientos como una barrera principal para la realización de pruebas de detección. En un estudio realizado con mujeres mayores de 40 años, el 99.2% citó la falta de información y educación como las razones para no someterse a una mamografía^(23)^. Una encuesta nacional reveló que el 13% de las mujeres que no acudían a las pruebas de detección desconocían la necesidad de la autoexploración mamaria y el 10% no sabía cómo realizarla. Se observan patrones similares en Oriente Medio y el norte de África: en Egipto, las mujeres que conocían y practicaban regularmente la autoexploración mamaria tenían una probabilidad significativamente menor de ser diagnosticadas en una fase avanzada^(24)^, mientras que en Irán, un estudio multicéntrico demostró que el conocimiento y la práctica de la autoexploración mamaria reducían el riesgo de diagnóstico en fase avanzada^(25)^. En Jordania, sin embargo, solo el 25.5% de las mujeres practicaban regularmente la autoexploración mamaria, el 10.5% se sometían a exploraciones mamarias clínicas periódicas y solo el 11.1% declaraban someterse a mamografías de forma sistemática^(8)^. Entre las barreras identificadas se encontraban las preocupaciones personales, la percepción de molestias relacionadas con el examen, la baja motivación por la salud, las creencias religiosas, las normas sociales, el apoyo social insuficiente, los temores relacionados con el estigma y las responsabilidades laborales y familiares que compiten entre sí. Si bien el nivel de educación se asociaba positivamente con el conocimiento, las limitaciones culturales y sociales a menudo impedían que la concienciación se tradujera en un comportamiento de detección sistemático. Estos hallazgos indican que mejorar el conocimiento por sí solo puede ser insuficiente a menos que las intervenciones aborden las normas culturales y los sistemas de apoyo social subyacentes.

Más allá de la concienciación individual, las barreras sistémicas, en particular los largos tiempos de espera para las citas médicas, se revelaron como un importante factor disuasorio para la participación en las pruebas de detección. En nuestro estudio, el 61.4% de las mujeres que nunca se habían sometido a pruebas de detección citaron los largos tiempos de espera como la razón principal. Para las poblaciones desfavorecidas, los retrasos prolongados y el acceso restringido a la atención médica agravan el riesgo de un diagnóstico tardío. Esto concuerda con investigaciones anteriores que muestran que las mujeres que tienen dificultades para acceder a la atención médica son menos propensas a participar en programas de detección, lo que en última instancia aumenta la probabilidad de que la enfermedad se presente en una etapa avanzada^(26)^. Abordar estas barreras requiere más que educación sanitaria; exige reformas estructurales, como el aumento de la capacidad de los servicios de detección, la reducción de los retrasos en las citas y la distribución equitativa de los recursos. Las intervenciones integrales dirigidas tanto a la alfabetización sanitaria como a la accesibilidad del sistema de salud son esenciales para reducir las disparidades y mejorar los resultados de la detección precoz.

La edad media de las participantes era de 44,5 ± 15,5 años, y síntomas como la retracción del pezón, los hoyuelos y las hendiduras, comúnmente asociados a estadios avanzados del cáncer de mama, eran significativamente más frecuentes entre las mujeres de más edad (p < 0,05). Este hallazgo demuestra que la edad no solo es un factor de riesgo, sino que también influye en la gravedad y la naturaleza de los síntomas. Un estudio identificó a las mujeres de entre 50 y 69 años como el grupo más afectado, con una incidencia que aumenta con la edad^(26)^. Además, el cáncer de mama en mujeres más jóvenes tiende a presentar características más agresivas y, a menudo, se diagnostica en una etapa más avanzada debido a los retrasos en su reconocimiento^(27)^. En África, la edad media en el momento del diagnóstico es de 48,7 años, inferior a la de los países de ingresos altos^(15)^. En los países de ingresos bajos y medios, la edad en el momento del diagnóstico tiende a ser más temprana^(16)^. Por ejemplo, en el África subsahariana oscila entre los 45 y los 52 años, en Oriente Medio entre los 36 y los 56 años, y en América Latina entre los 49 y los 53 años. En China, la mayor carga de morbilidad se observa en el grupo de edad de 50 a 59 años, con un marcado aumento de la incidencia entre las mujeres mayores de 40 años^(13)^. Del mismo modo, en los países de ingresos altos, el riesgo más elevado se da entre las mujeres de 50 años o más, y sigue aumentando en este grupo de edad^(12)^. Un gran metaanálisis multicéntrico de 44 estudios informó una edad media de diagnóstico que oscilaba entre los 55 y los 76,4 años, con un aumento significativo del riesgo después de la menopausia^(3)^. En Brasil, la edad media en el momento del diagnóstico fue de 55 años^(17)^. En conjunto, estos hallazgos demuestran que la edad influye no solo en el nivel de riesgo de cáncer de mama, sino también en la presentación de los síntomas, la progresión de la enfermedad y el momento del diagnóstico. Por lo tanto, los programas de detección deben incorporar tanto la edad como las características de los síntomas en la estratificación del riesgo.

La menarquia precoz y la menopausia tardía, indicativas de una exposición prolongada al estrógeno, se consideran factores de riesgo significativos para el cáncer de mama^(28,29)^. Las investigaciones han demostrado que la menarquia después de los 15 años reduce el riesgo de cáncer de mama en un 23% en comparación con la menarquia antes de los 12 años^(30)^. Los estudios sobre los factores hormonales indican que la exposición prolongada al estrógeno aumenta el riesgo de cáncer de mama, una afección que se observa típicamente en mujeres con inicio precoz de la menstruación (< 12 años) y menopausia tardía (≤ 55 años)^(12,18)^. Además, no tener hijos o tener el primer hijo después de los 30 años también se considera un factor de riesgo adicional para el cáncer de mama^(12)^. Aunque nuestro estudio encontró tasas más altas de síntomas de cáncer de mama entre las mujeres con menarquia precoz y menopausia tardía, las diferencias no fueron estadísticamente significativas (p > 0,05). Esto puede deberse al tamaño limitado de la muestra en el grupo de menopausia tardía o a la distribución general por edades. Sin embargo, el valor p para la menopausia tardía (p = 0,065) se acercó a la significación estadística, lo que sugiere una tendencia clínicamente significativa. Estos resultados coinciden con la bibliografía existente, que indica que la exposición hormonal prolongada puede aumentar la probabilidad de síntomas de cáncer de mama.

Nuestro estudio examinó factores reproductivos como la edad en el primer parto, el número de embarazos y el número de hijos vivos en relación con la presencia de síntomas de cáncer de mama. Aunque la tasa de síntomas fue menor entre las mujeres que dieron a luz antes de los 30 años, la diferencia no fue estadísticamente significativa (p < 0,05). No obstante, la bibliografía existente respalda de manera consistente el efecto protector del parto precoz y la alta paridad contra el cáncer de mama. Por ejemplo, se ha demostrado que las mujeres que dieron a luz antes de los 30 años tienen un menor riesgo de desarrollar subtipos luminales de cáncer de mama^(28)^. Del mismo modo, el primer parto antes de los 20 años se considera protector, mientras que el parto después de los 35 años se considera un factor de riesgo^(30)^. Se cree que el mecanismo protector implica la regulación hormonal durante el embarazo y la reducción de la exposición al estrógeno. También se ha descubierto que un mayor número de embarazos reduce el riesgo de cáncer de mama. Las mujeres que nunca dan a luz tienen un mayor riesgo, que se acentúa cuando el primer embarazo se produce a una edad más avanzada. Aunque en nuestro estudio no se encontraron diferencias estadísticamente significativas, estos hallazgos subrayan la importancia de controlar los factores reproductivos en relación con los síntomas del cáncer de mama y ponen de relieve la necesidad de realizar estudios con muestras de mayor tamaño.

Un hallazgo significativo de nuestro estudio fue que las mujeres que amamantaron durante menos de 24 meses eran más propensas a reportar síntomas de cáncer de mama (p < 0,05). Este hallazgo respalda el papel protector de la lactancia materna contra el cáncer de mama. La duración y el momento de la lactancia materna, junto con la paridad, pueden contribuir a este efecto protector. Una revisión sistemática encontró que la lactancia materna a largo plazo proporciona una protección significativa contra varios subtipos de cáncer de mama^(31)^. Otro estudio destacó que la lactancia materna favorece la regulación hormonal y la diferenciación de las glándulas mamarias, lo que ayuda a prevenir el cáncer^(28)^. Un metaanálisis mostró que cada periodo de 12 meses de lactancia materna reduce el riesgo de cáncer de mama en un 4,5%, y cada parto adicional reduce el riesgo en aproximadamente un 7%^(32)^. Otro estudio informó de que la lactancia materna puede reducir el riesgo de cáncer de mama hasta en un 16%^(33)^. El efecto protector es especialmente pronunciado entre las mujeres que dieron a luz a una edad temprana y amamantaron durante períodos más largos. El hecho de que las mujeres con síntomas de nuestro estudio amamantaran durante menos de 24 meses concuerda con esta bibliografía. Por lo tanto, la lactancia materna desempeña un papel fundamental no solo en la salud del bebé, sino también en la reducción del riesgo de cáncer de mama a largo plazo. Los programas de concienciación y apoyo que promueven períodos de lactancia materna más largos pueden aportar importantes beneficios para la salud, tanto a nivel individual como poblacional.

Nuestro estudio no encontró una asociación estadísticamente significativa entre la terapia hormonal o el uso de anticonceptivos hormonales y la presencia de síntomas de cáncer de mama (p > 0,05). Sin embargo, la literatura existente sugiere que la exposición hormonal puede aumentar el riesgo de cáncer de mama. Por ejemplo, la terapia de reemplazo hormonal (TRH) posmenopáusica se asocia con un riesgo elevado. Un estudio realizado en el Reino Unido informó un riesgo relativo de 1,66 de cáncer de mama entre las usuarias de TRH^(34)^. Otro estudio descubrió que el uso prolongado de la terapia combinada de estrógenos y progestágenos aumentaba significativamente el riesgo, que disminuía tras la interrupción^(35)^. Los anticonceptivos hormonales también se han asociado con un mayor riesgo de cáncer de mama, especialmente con el uso prolongado. Un amplio estudio de cohorte informó de que el uso de anticonceptivos hormonales aumentaba el riesgo de cáncer de mama en 1,20 veces y que existía una relación dosis-respuesta basada en la duración^(36)^. Otro estudio identificó el uso de anticonceptivos orales como un factor de riesgo significativo (OR = 1,16)^(33)^. La falta de hallazgos significativos en nuestro estudio puede deberse a la falta de datos sobre la duración y el tipo de terapia hormonal utilizada. Estos hallazgos subrayan la importancia de los enfoques de prevención y tratamiento personalizados que tienen en cuenta los factores de riesgo individuales y los antecedentes de exposición hormonal.

La obesidad, la dieta, el tabaquismo y el consumo de alcohol se encuentran entre los factores relacionados con el estilo de vida que, según estudios epidemiológicos de cohortes y de casos y controles, influyen en el riesgo y el pronóstico del cáncer de mama^(18)^. Cada vez hay más pruebas que respaldan la relación entre el tabaquismo y el riesgo de cáncer de mama. Una revisión sistemática demostró que fumar aumenta significativamente el riesgo de cáncer de mama y que el riesgo aumenta proporcionalmente a la cantidad fumada^(37)^. La exposición temprana al tabaquismo puede provocar cambios en el tejido mamario. Los estudios han encontrado una asociación significativa entre el inicio temprano del tabaquismo y el riesgo de cáncer de mama^(38)^. Las adolescentes que fuman, especialmente aquellas con antecedentes familiares de cáncer de mama, corren un mayor riesgo^(39)^. Otras investigaciones han demostrado que la nicotina altera el microambiente tumoral, favorece la metástasis y contribuye a la resistencia a la quimioterapia^(40,41)^. El efecto del tabaquismo sobre el cáncer de mama varía según la frecuencia, la duración del abandono, el estado de fertilidad y los subtipos de cáncer^(42)^. Sin embargo, en nuestro estudio no se observó una relación significativa entre el tabaquismo y los síntomas del cáncer de mama. Este resultado puede deberse a la falta de datos sobre la duración del tabaquismo, la intensidad, la exposición pasiva o la variabilidad en el reconocimiento de los síntomas entre las participantes.

No obstante, es importante interpretar estos hallazgos a la luz de ciertas limitaciones. Este estudio se llevó a cabo en una sola ciudad, lo que podría limitar la generalización de los resultados a otras regiones de Turquía. La muestra solo incluyó participantes mujeres; por lo tanto, los hallazgos no pueden generalizarse a los hombres, y no se recopilaron datos sobre el cáncer de mama masculino, aunque es poco frecuente. Además, dado que los datos fueron autoinformados, el sesgo de recuerdo y de notificación podría haber influido en los resultados.

## CONCLUSIÓN

Este estudio demostró que los síntomas del cáncer de mama son frecuentes entre las mujeres y que la participación en los programas de detección sigue siendo insuficiente. Los síntomas fueron reportados con mayor frecuencia por mujeres que amamantaron durante menos tiempo, carecían de conocimientos sobre la autoexploración mamaria y tenían un acceso limitado a los servicios de salud. Se observó que factores como la edad, la duración de la lactancia materna y la conciencia individual influían significativamente en la aparición de los síntomas. Aunque no se observaron asociaciones estadísticamente significativas entre los factores hormonales o los antecedentes reproductivos y la presencia de síntomas, las tendencias identificadas sugieren patrones clínicamente relevantes. Estos resultados subrayan la necesidad de mejorar las iniciativas de conciencia-ción, garantizar servicios de detección accesibles y oportunos, y desarrollar estrategias preventivas adaptadas a los perfiles de riesgo individuales en la lucha contra el cáncer de mama.

## Data Availability

El autor puede facilitar el archivo de datos por correo electrónico previa solicitud razonable de los lectores.
